# Towards effective cystic fibrosis gene therapy by optimizing prime editing and pulmonary-targeted LNPs

**DOI:** 10.3389/fsysb.2025.1603749

**Published:** 2025-12-03

**Authors:** Kaya Sophie Lange, Lisa Marie Wiesner, Kathleen Susat, Vera Köhler, Malte Lenger, Christian Alexander Michalek, Anna-Lena Baack, Philip Frederic Mundt, Kai Kanthak, Isabell Alexandra Guckes, Liliana Sanfilippo, Lucas Haverkamp, Utkarsh Anil Mahajan, Felicitas Helena Zimmer, Sinan Zimmermann, Marco Tobias Radukic, Levin Joe Klages, Jörn Kalinowski, Kristian Mark Müller

**Affiliations:** 1 iGEM Bielefeld-CeBiTec, Center for Biotechnology, Bielefeld University, Bielefeld, Germany; 2 Cellular and Molecular Biotechnology, Faculty of Technology, Bielefeld University, Bielefeld, Germany; 3 Proteome and Metabolome Research, Faculty of Biology, Bielefeld University, Bielefeld, Germany; 4 Microbial Genomic and Biotechnology, Center for Biotechnology, Bielefeld University, Bielefeld, Germany

**Keywords:** cystic fibrosis, gene therapy, prime editing, gene editing, lipid nanoparticles, mucociliary clearance, *in vitro* prime editing

## Abstract

Cystic fibrosis (CF) is the most prevalent inherited disease. Inactivating mutations in the Cystic Fibrosis Transmembrane Conductance Regulator (CFTR) gene lead to the accumulation of viscous mucus and subsequent respiratory complications. This study optimized a prime editing (PE) approach to correct CFTR mutations focusing on the F508del mutation. Prime editing allowed to introduce missing bases without double-strand breaks using a Cas9-nickase fused with a reverse transcriptase in combination with a prime editing guide RNA (pegRNA). Various self-designed pegRNAs were compared. For delivery, various lipid nanoparticles (LNP) were tested, which were optimized for stability and lung cells targeting based on lipid selection or chitosan complexion. A fluorescence reporter system, pPEAR_CFTR, was developed mimicking F508del for validation. The five pegRNAs yielding the highest efficiency were used for genomic CFTR correction in a CF bronchial cell line. Nanopore sequencing of genomic DNA revealed approximate 5% edited reads. These results highlight the promise of prime editing-LNP systems for precise and lung-specific gene correction, paving the way for novel therapies in cystic fibrosis and other pulmonary genetic disorders.

## 1 Introduction

Cystic Fibrosis (CF) is a life-limiting, inherited, genetic disorder. The European Cystic Fibrosis Society Patient Registry indicates a median age at death of 33 years for individuals with CF in Europe, highlighting the severe and life-shortening nature of this disorder (Zolin et al., 2024). Recent data also show improvements in lung function and life expectancy, reflecting the impact of advances in care and treatment (Kerem et al., 2024). The cause of CF are mutations in the Cystic Fibrosis Transmembrane Conductance Regulator (*CFTR*) gene, which impair the function of the CFTR protein through various mechanisms such as misfolding, defective processing or reduced channel activity (De Boeck, 2020). The protein acts as an ion channel that regulates chloride ion movement across epithelial cell membranes in various tissues (Hwang and Kirk, 2013). This ion flow is essential for maintaining water homeostasis through osmotic gradients, ensuring adequate mucus hydration and consistency, which is crucial for the body’s natural defense mechanism of trapping and clearing pathogens and other inhaled particles via mucociliary clearance.

**FIGURE 1:**
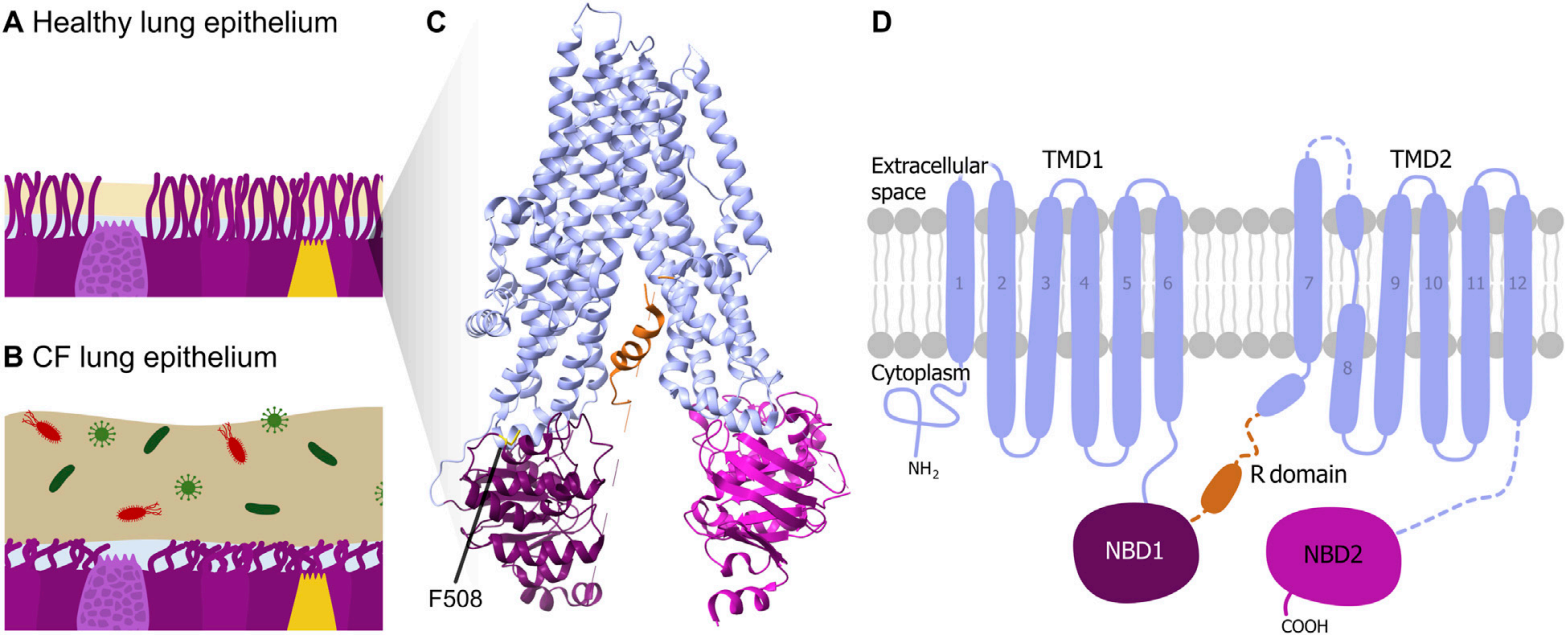
CF pathophysiology and CFTR structure. Schematic illustration of lung epithelial tissue and mucus of **(A)** a healthy person and of **(B)** a CF patient. **(C)** 3D structure and **(D)** schematic 2D domain structure of the CFTR protein according to (Liu et al., 2017) with the pore-forming transmembrane domains TMD1 and TMD2 (light blue), the nucleotide-binding domains NBD1 (purple) and NBD2 (pink), and the (regulatory) R domain (orange). The phenylalanine amino acid at position 508 (F508) is indicated in the 3D structure.

To date more than 2,000 mutations in the *CFTR* gene are known, with the F508del mutation NM_000492.3(CFTR):c.1521_1523del (p.Phe508del) being the most common, affecting about 70% of CF patients (De Boeck, 2020; Rodrigues et al., 2008). This mutation involves the deletion of three nucleotides (*CTT*), leading to the loss of a phenylalanine residue at amino acid position 508 (Amico et al., 2019). This deletion impairs the kinetic and thermodynamic folding of the Nucleotide Binding Domain (NBD) 1 domain of the protein (Figure 1), resulting in not only misfolds but also defects in trafficking and premature degradation, leading to a reduction in surface protein expression (Amico et al., 2019). Therefore, the mucus thickens abnormally, obstructing airways and digestive ducts, which results in various symptoms like chronic lung infections by impaired mucociliary clearance, inflammation, digestive impairment, and malnutrition (Haack, 2013).

Due to the so far incurable nature of the disease, treatment strategies typically aim to improve the symptoms through multiple approaches, including lifelong daily medications, physical therapy, and dietary adjustments (Bierlaagh et al., 2021). One of the most significant advances in CF treatment has been the development of CFTR modulators, which temporarily prevent protein misfolding (Wainwright et al., 2015) and can improve overall health in many patients. However, they do not address the underlying genetic cause and are not available to every patient due to high costs (De Boeck, 2020; Lee et al., 2020). Gene editing approaches, particularly prime editing, offer a promising alternative by directly correcting disease-causing mutations at the DNA level rather than merely mitigating symptoms. Gene therapy for CF has advanced significantly since the 1990s, when early trials using viral vectors faced immune responses and poor gene delivery efficiency (Lomunova and Gershovich, 2023). Recent developments center around mRNA-based therapies and gene editing showing encouraging preclinical results and leading to clinical trials (Choi and Engelhardt, 2021). The current focus is improving lung-targeted delivery using optimized vectors and nanoparticles to achieve lasting *CFTR* correction with minimal immune response (First inhaled lentiviral gene therapy enters cystic fibrosis trial, 2025). Nevertheless, there is still no approved gene therapy for CF (Lomunova and Gershovich, 2023), demonstrating the need for further research.

**FIGURE 2:**
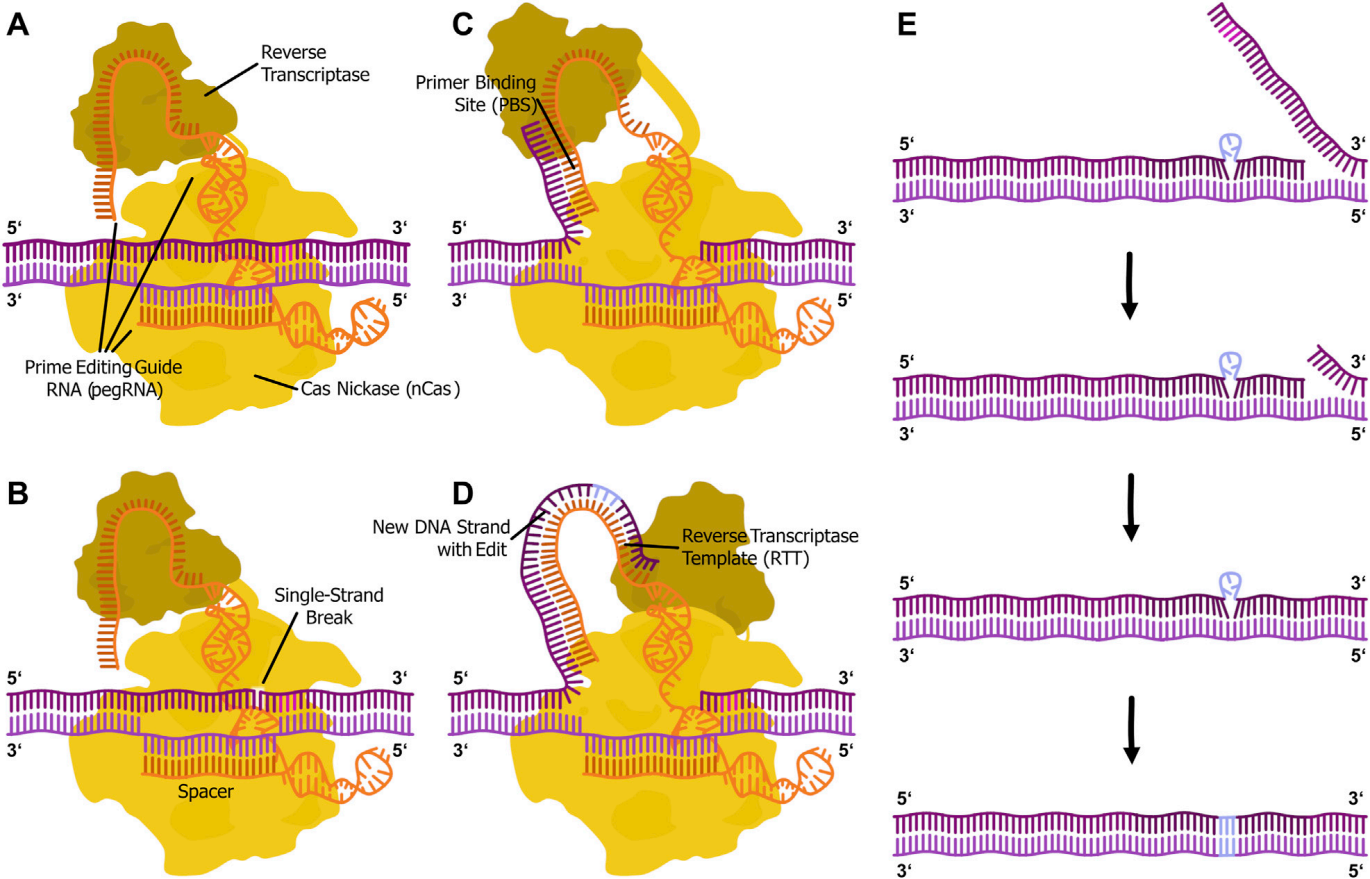
Illustration of the prime editing mechanism. **(A)** The prime editor fusion protein (PE with Cas nickase domain (yellow) and reverse transcriptase domain (ochre)) complexed with a prime editing guide RNA (pegRNA with spacer, primer binding site and reverse transcriptase template (orange)) locates the genomic DNA (purple) target via DNA-RNA duplex formation and PAM (magenta) recognition by the Cas protein. **(B)** The nickase introduces a single-strand break in the PAM-containing strand. **(C)** The primer binding site (PBS) of the pegRNA binds to the 3′-flap of the cut DNA strand. **(D)** The reverse transcriptase synthesizes the new DNA strand, including the intended edit (light blue), in 5′ to 3′ direction from the primer based on the reverse transcriptase template (RTT) in the pegRNA. **(E)** The 3′ flap binds back to the genome. This step happens by chance, since both flaps are in an equilibrium. The 5′ overlap is excised by cellular exonucleases. The two flaps are ligated. Cellular mismatch repair integrates the edit into the genome.

A promising approach for the targeted correction of *CFTR* mutations is prime editing, a precise genome-editing technique that enables targeted nucleotide modifications without inducing double-strand breaks (Figure 2). It relies on a fusion protein consisting of a catalytically impaired Cas-based nickase (nCas) and a reverse transcriptase (RT). The system is guided to a specific DNA sequence in the genome by a prime editing guide RNA (pegRNA), which also provides a template for the desired sequence modification. At the target DNA sequence, nCas introduces a single strand break in the non-target strand, creating an entry point for the reverse transcription process. The RT synthesizes a complementary DNA sequence, facilitating the incorporation of the intended genetic modification. The cellular repair mechanisms subsequently integrate the newly synthesized DNA into the genome, completing the editing process (Anzalone et al., 2019).

To effectively implement prime editing for *CFTR* correction in lung epithelial cells, an efficient and targeted delivery system is essential. Multiple delivery systems have been explored, with Lipid-Nanoparticles (LNPs) being the most promising option due to their lower immunogenicity (Verma et al., 2023) and greater loading capacity (Taha et al., 2022) for DNA and RNA, compared to other vectors, such as adeno-associated viruses (AAVs). The small size (60–200 nm) and biocompatibility of LNPs make them crucial for gene therapy and mRNA-based vaccines (Simonsen, 2024). Once in the body, LNPs are taken up by cells through endocytosis, but their therapeutic success relies on efficient endosomal escape. Deficiencies in endosomal escape increase vulnerability of mRNA to lysosomal degradation, compromising treatment effectiveness (Wang et al., 2024). To maximize therapeutic efficiency, we utilized the selective organ targeting (SORT) strategy (Cheng et al., 2020) to systematically engineer an LNP formulation optimized for lung-specific delivery. Compared to conventional LNP formulations consisting of ionizable lipids, phospholipids, pegylated lipids and cholesterol, an additional component, the SORT molecule, is integrated whose organ specificity is determined by the molecular chemistry and percentage in the LNP formulation (Cheng et al., 2020).

In this study, we optimized a prime editing strategy targeting the F508del mutation in *CFTR* by designing and testing tailored pegRNAs. We then delivered them alongside prime-editing mRNA via a lung-targeted LNP formulation into a human bronchial cell line, carrying the F508del mutation. Furthermore, we established a novel Prime Editing Activity Reporter (PEAR)-based model system to evaluate the prime editing efficiency *in vitro*. By correcting the F508del mutation, we aim to address the genetic cause of Cystic Fibrosis and restore CFTR function.

## 2 Materials and methods

### 2.1 Cell culture

The HEK293 cell line was obtained from Leibniz-Institute DSMZ (Germany), while the 508del carrying cell line was a kind gift of Prof. Dr. Ignatova from Hamburg University. Both cell lines were regularly tested for the presence of *mycoplasma* (Venor GeM Advance, Minerva Biolabs, Germany) and found to be negative. To verify that the F508del carrying cell line carries the expected homozygous mutation, we sequenced the genomic region surrounding the mutation via Nanopore sequencing. The results showed that the cells were not homozygous for the F508del mutation as 83% of sequenced cells showed no mutation in the expected region. Besides this mutation, the V470M mutation was also detected, which the cell line appears to be homozygous for. In the following, the partly F508del carrying cell line is referred to as CFBE-X.

HEK293 and CFBE-X cell lines were cultivated in DMEM (Capricorn Scientific, Germany), which was supplemented with 10% (v/v) fetal bovine serum (StemCell Technologies, Germany) and 1% penicillin-streptomycin (P/S; Capricorn Scientific, Germany). The cells were maintained at 37 °C in a 5% CO_2_ atmosphere (standard cultivation conditions). The cultures were passaged every 3–4 days upon reaching 90% confluency using Trypsin/EDTA (StemCell Technologies, Germany).

### 2.2 Transfection

The cells were seeded on 24-well plates (Sarstedt, Germany) at a density of 5 × 10^4^ cells/well and transfected after 24 h. The fluorescent protein mVenus expressing plasmid pZMB0938 (Supplementary Table S1) was used as positive control for the transfection process and untreated cells were classified as negative control. The total amount of plasmid used with Lipofectamine transfection was 500 ng, comprising 50 ng of PEAR target plasmid, 150 ng of pegRNA, and 300 ng of prime editor (PE) coding plasmid adapted from Simon et al. (2022). In all experiments, the pCMV-PE6c (Simon et al., 2022; Bulcaen et al., 2024) plasmid was used as the PE expressing plasmid. The Lipofectamine transfection process was carried out in accordance with the manufacturer’s guidelines, utilizing Lipofectamine 3000 (Thermo Fisher Scientific, Germany).

For transfection with LNPs, 50 µL per well of the LNP solution containing 1,000 ng RNA was slowly dripped onto the cells in a 24-well plate in DMEM containing 10% FBS as well as 1% P/S and then cultivated under standard conditions.

For the transfection process using Polyethylenimine (PEI), a fresh mixture was prepared. For each well, 1,000 ng of plasmid DNA were mixed 100 µL of serum-free Opti-MEM. Subsequently, PEI (Thermo Scientific, Germany) at a concentration of 0.5 μg/μL was added at a ratio of 1:4 (i.e. 400 µL of PEI). The mixture was gently stirred and incubated at room temperature for 10–20 min to allow PEI to bind DNA. The cells were then washed with phosphate buffered saline (NaCl 137 mM, KCl 2.6 mM, Na_2_HPO_4_ 10 mM, KH_2_PO_4_ 1.8 mM; pH 7-7,4) to remove excess serum. The PEI-DNA-Opti-MEM mixture was added directly to the cells prior to incubation with the transfection mixture at 37 °C for 4–6 h. After incubation, the mixture was removed and replaced with DMEM.

The cells were analyzed 72 h post transfection by fluorescence microscopy (Leica DMI6000 B at 20× magnification, Leica Microsystems, Germany) to assess their viability. All cells were washed with phosphate buffered saline and detached with Accutase (Capricorn, Germany) for 10 min under standard cultivation conditions. The cells were analyzed by flow cytometry with BD Accuri C6 Plus flow cytometer (BD Biosciences, United States). For each sample, typically 1 × 10^6^ events were measured by exciting at 488 nm and measuring FL1 channel (533/30 nm). Events which displayed a fluorescence higher than 99% of viable cells of the control were counted as fluorescent cells. Analysis was carried out using FlowJo 10.9 (BD Biosciences, United States).

### 2.3 Plasmid construction

All plasmids used for cloning were purchased from Addgene (Supplementary Table S1). The PEAR_CFTR plasmid (pDas12124_PEAR-GFP-F508del) was constructed by digesting pDas12124_PEAR-GFP-preedited (Addgene: 177179) (Simon et al., 2022) with NheI (NEB, Germany) and Xhol (NEB, Germany), followed by Gibson cloning of the modified sequence mimicking the genomic target site (GFP-GGTdel) kindly provided as gene synthesis from Integrated DNA Technologies (United States). Digestions and Gibson cloning were done according to NEBcloner and Gibson Assembly Master Mix-Assembly (E2611) protocols from NEB (Germany). The pegRNA expression vectors were created by golden gate cloning of annealed oligonucleotides into the pU6-pegRNA-GG-acceptor plasmid (Addgene: 132777) according to the cloning protocol established by Anzalone et al., 2019. Correct cloning of all plasmids was validated through Sanger sequencing of the cloning sites. All oligonucleotides for pegRNA construction, primers for colony PCR and fragment amplification as well as gene syntheses are listed in Supplementary Tables S1–S3.

### 2.4 Design of pegRNA variants

The web tool pegFinder (Chow et al., 2021) was used to create the first versions of pegRNAs which were then refined rationally. The choice of spacer, the basis for pegRNA design, was aided by the software CRISPick (Doench et al., 2016), which was used to evaluate off-target sites and cleavage potential.

### 2.5 Genomic DNA extraction and CFTR specific sequencing

Genomic DNA was extracted from the CFBE-X cell line using the NEB Monarch Genomic DNA purification Kit according to the manufacturer’s instructions. The extracted genomic DNA was subjected to PCR amplification using Taq DNA Polymerase (NEB, Germany) according to protocol and specific primers (Supplementary Tables S2 and S3) for the CFTR region of interest. The amplified PCR products were purified using NucleoSpin Gel and PCR clean-up (Macherey-Nagel, Germany) according to the manufacturer’s instructions. The purified products were then subjected to Nanopore sequencing using a ligation sequencing kit (SQK-NBD114.96). Long read data were produced on R10.4.1 flowcells (ONT) and basecalled with Dorado v. 7.6.7 in super-accuracy mode. The resulting reads were mapped to a reference sequence using minimap2 (Li, 2018) and visualized using IGV (Thorvaldsdottir et al., 2013) to confirm and quantify the presence of the F508 sequence.

### 2.6 RNA synthesis

Prior to RNA synthesis, the plasmid pCMV-PE6c was linearized using the restriction enzyme PvuI (NEB, Germany) in 10× CutSmart Buffer (NEB, Germany) for 3 h at 37 °C. After 2.5 h, the reaction was treated with Antarctic Phosphatase with corresponding buffer (NEB, Germany). Successful digestion was confirmed via analytical agarose gel electrophoresis, followed by purification of the linearized DNA using the NEB Monarch DNA purification Kit, with elution in 20 µL of ultrapure water. Concentration and purity of the treated DNA were assessed using spectrophotometry (Nanodrop 2000, Thermo Scientific, Germany). For RNA synthesis, the High ScribeT7 High Yield RNA Synthesis Kit (NEB, Germany) was used, along with a 3′-O-Me-m7G (5′)ppp (5′)G cap analog (NEB, Germany). The reaction was incubated at 37 °C for 1.5 h. ATP, UTP, and CTP were added at a final concentration of 5 mM, and GTP at a final concentration of 1 mM and the incubation continued at 37 °C for 1.5 h. Following synthesis, a DNase I (NEB, Germany) digestion step was performed, according to the NEB protocol, to remove residual DNA. The RNA was then subjected to poly(A) tailing using the NEB Poly(A) Polymerase Kit to enhance transcript stability and translational efficiency. Post-reaction purification was carried out with the Monarch RNA clean up Kit (NEB, Germany). The RNA concentration and purity were determined via spectrophotometry (Nanodrop 2000, Thermo Scientific, Germany).

### 2.7 RNA chitosan complexation

Chitosan with a viscosity of 5 centipoise and ultra-low molecular weight (Glentham Life Sciences, Germany) was prepared by dissolving 1 g in 100 mL of 1% acetic acid overnight, followed by autoclaving. The pH was adjusted to 5.5 using NaOH, and dilutions of 5,000 ng/μL and 2,000 ng/μL were prepared. For chitosan complex formation, RNA and chitosan solution were each heated to 50 °C. Then pegRNA (5,000 ng/μL) with chitosan (5,000 ng/μL) and the PE6c RNA (2,000 ng/μL) with chitosan (2,000 ng/μL) were mixed at a ratio of 1.5:1 (RNA volume: chitosan volume) by pipetting ten times.

### 2.8 LNP formulation

The LNP formulation process consisted of two main steps: preparation of the lipid mixture and formation of the LNPs with the RNA-chitosan complexes. Lipid stock solutions were prepared in 99% ethanol with the following final concentrations: 4A3-SC8 (Echelon Bioscience, United States) 150 mg/mL, DOPE (1,2-di-(9Z-octadecenoyl)-sn-glycero-3-phosphoethanolamin, Avanti Polar Lipids, United States) 10 mg/mL, cholesterol (Cayman, United States) 10 mg/mL, mPEG-2000-DSPE, Na (sodium;[(2R)-2,3-di(octadecanoyloxy)propyl] 2-(2-methoxyethoxycarbonylamino)ethyl phosphate, Corden Pharma, Switzerland) 10 mg/mL, and DOTAP (1,2-dioleoyl-3-trimethylammonium-propane, Corden Pharma, Switzerland) 32.4 mg/mL. In a separate formulation, a plant-based cholesterol, BotaniChol (Corden Pharma, Switzerland), was used at the same concentration (10 mg/mL) as the standard cholesterol. The final lipid mixture was prepared by combining 6.7 μL 4A3-SC8 solution, 50.7 μL DOPE solution, 52.7 μL cholesterol or BotaniChol solution, 34.2 μL mPEG-2000-DSPE solution, and 40.0 μL DOTAP, resulting in a molar ratio of 14.5:14.5:28.9:2.7:39.4, followed by mixing until a clear solution was obtained.

For RNA mixture formation, the PE6c RNA-chitosan complex and the pegRNA-chitosan complex were combined at a 2:1 (m:m) in 8 μL and added to 52 μL citrate buffer (10 mM, pH 4; Thermo Scientific, Germany). In the next step 19.44 μL lipid mixture were combined with 0.66 μL 99% ethanol and the RNA mixture was rapidly and pipetted up and down for 30 s. The final ratio of aqueous to ethanol solution was 3:1 (v:v). LNPs were then incubated for 15 min at room temperature, followed by overnight dialysis against 1× phosphate buffered saline to remove ethanol and acidic buffer.

### 2.9 Physicochemical characterization of LNPs

Size and size distribution of the LNPs were assessed using Zetasizer measurements and cryo-transmission electron microscopy (cryo-TEM). Zetasizer analysis (Microtrac, Germany) included dynamic light scattering (DLS) to determine particle size distribution, polydispersity index (PDI) as a measure of size homogeneity, and zeta potential to evaluate surface charge. For these measurements, 600 µL of LNP solution was analyzed. Dialysis was performed in ultrapure water instead of phosphate buffered saline to reduce background charge and minimize any interference with the measurements. For structural analysis, LNP samples were vitrified on holey carbon TEM grids (Lacey Carbon Film, 200 mesh; Science Services, Germany) using a Leica EM GP Blotting and Plunging System (Leica, Germany). The grids were rapidly immersed in liquid ethane, cooled with liquid nitrogen, ensuring ultra-fast vitrification. Samples were then transferred to a cryo-transfer and tomography holder (Fischione Model 2550, E.A. Fischione Instruments, United States) to maintain cryogenic conditions during imaging. Image acquisition and processing were conducted using Digital Micrograph GMS 3 (Gatan, United States).

### 2.10 Cytotoxicity assay

In preparation for the cytotoxicity assay, 1 × 10^4^ CFBE-X cells per well were seeded in a 96-well-plate in 50 μL cell culture medium. Background fluorescence was measured using triplicates of 100 µL medium. For evaluating the cytotoxicity of LNPs without cargo, serial dilutions at different concentrations were prepared, followed by the addition of 50 µL of the LNP solution into the well plate. The samples were incubated for 24 h at 37 °C. After incubation, the samples were treated with 10 µL of 0.1 g/L resazurin stock solution. Measurements were performed using a microplate reader (Microplate-reader Infinite M Plex, Tecan, Switzerland) at an excitation wavelength of 545 nm, and an emission wavelength of 590 nm. Data collection took place several times over 4 h.

### 2.11 Calculation of prime editing efficiency

To determine prime editing efficiency, two different approaches were used depending on the system applied. In the reporter plasmid system (pPEAR_CFTR), prime editing restores green fluorescent protein (GFP) fluorescence upon successful editing of a mutated splice site. The editing efficiency was calculated by normalizing the fluorescence signal of edited cells to that of cells transfected with a GFP-expressing control plasmid, which represents the maximum achievable fluorescence signal under our transfection conditions.

$$prime\;editing\;efficiency\;\left(1\right)=\frac{\%\;Fluorescent\;Cells\;\left(edited\right)}{\%\;Fluorescent\;Cells\;\left(GFP\;control\right)}\times 100$$

This calculation provides a relative measure of editing efficiency, contextualized to the maximum observable GFP signal. It accounts for variability in transfection efficiency and allows comparison between different pegRNAs.

### 2.12 Statistical analysis

Unless otherwise described, all statistical analyses were performed using Prism 5 (GraphPad Software, United States) and one-way ANOVA, followed by Tukey’s *post hoc* test to determine significant differences (p < 0.05). Data is presented as mean ± standard error (SE), with n = 3. Identical letters indicate groups that are not significantly different, whereas different letters represent statistically distinct datasets. Background noise was accounted by corresponding negative and positive controls as described in the figure legends.

## 3 Results

### 3.1 PegRNA architecture with 16 nt PBS, 33 nt RTT and no silent edits enhances prime editing efficiency with pPEAR_CFTR in CFBE-X

**FIGURE 3:**
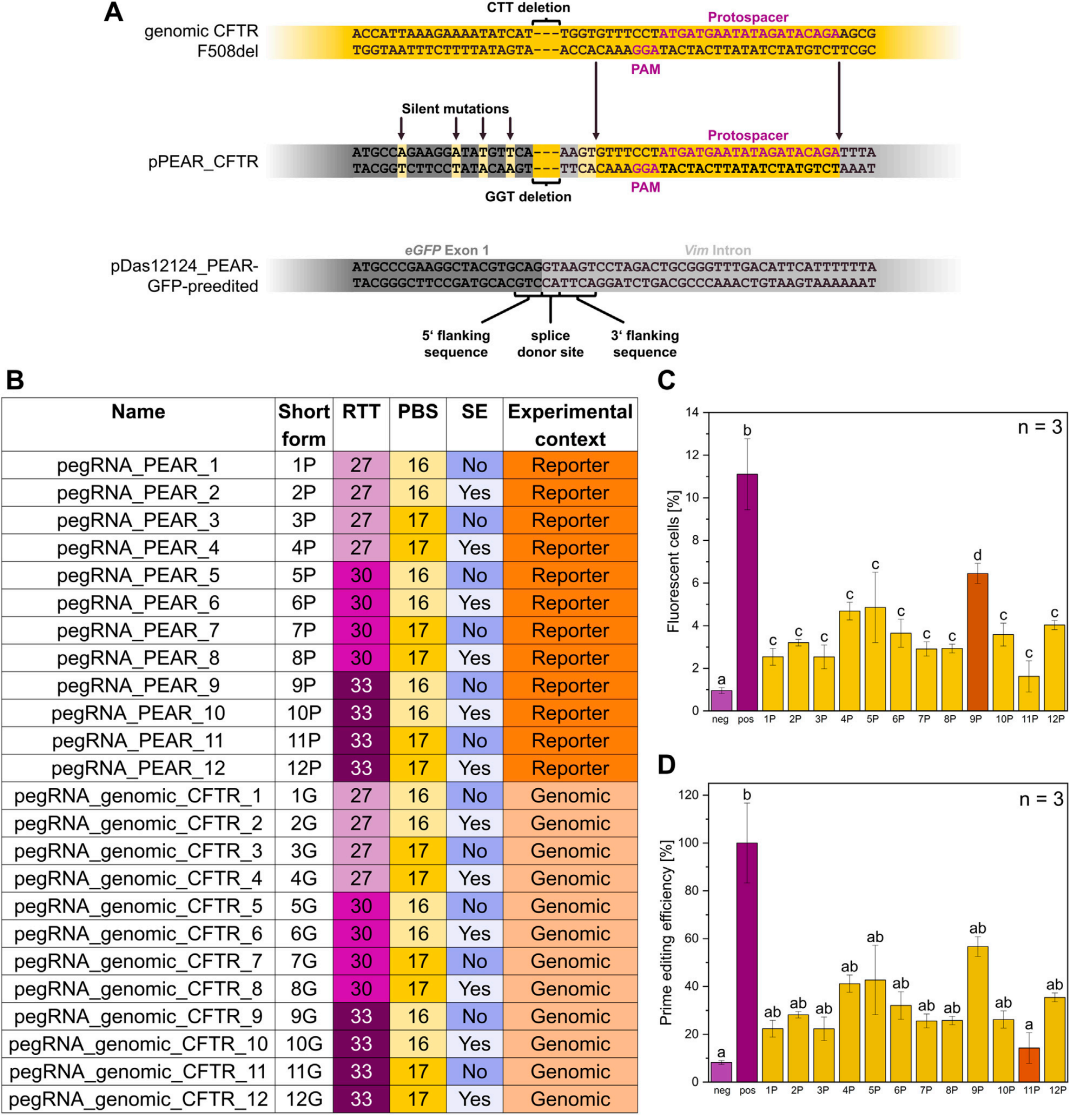
Features of the reporter plasmid pPEAR_CFTR. **(A)** Excerpts of the sequences of genomic CFTR F508del, the pPEAR_CFTR plasmid constructed and used in this study and the pDas12124_PEAR-GFP-preedited plasmid (Simon et al., 2022), respectively. pPEAR_CFTR differs from pDas12124_PEAR-GFP-preedited in three aspects: A 3 bp deletion at the splice donor site resembling the deletion found in the CFTR F508del sequence, a 27 bp substitution 4 bp downstream of the deletion derived from the genomic CFTR sequence and 4 silent mutations upstream of the deletion increasing the AT content. The upper sequence of each sequence pair represents the coding DNA strand, and the lower sequence represents the template strand. **(B)** Overview of all pegRNAs with name, short form, Reverse Transcriptase Template (RTT, light purple = 27 bp, purple = 30 bp, dark purple = 33 bp), Primer Binding Site (PBS, light yellow = 16 bp, dark yellow = 17 bp), Silent Edits (SE, dark blue = no, light blue = yes), and experimental context (dark orange = reporter, light orange = genomic). **(C)** Flow cytometry measurements of CFBE-X cells co-transfected with pPEAR_CFTR, pCMV-PE6c and pegRNA_PEAR variants, n = 3 biological replicates. Comparison of mean values of different pegRNAs varying in their architecture regarding the PBS, RTT, and incorporation or not of silent edits. The negative control (light purple) consists of untreated cells, the positive control (purple) includes cells transfected with a GFP expressing plasmid. Statistics were performed via one-way ANOVA with Tukey’s post hoc test. Within each graph, samples that differ significantly are indicated with a different letter and color (p < 0.05). **(D)** Prime editing efficiency of pegRNA_PEAR variants with pPEAR_CFTR, the data from **(C)** were normalized to the positive control, which was set to 100%, n = 3 biological replicates. Statistics were performed via one-way ANOVA with Tukey’s post hoc test. Within each graph, samples that differ significantly are indicated with a different letter and color (p < 0.05).

In this work, the fluorescence-based PEAR system, introduced by Simon et al., in 2022, was contextualized to resemble the prime editing target site of genomic *CFTR*. The PEAR system is based on a GFP coding sequence, that is divided into two pieces by an intron from the mouse *Vim* gene. The splice donor site and the surrounding region are mutated in a way that disables splicing, thus preventing correct GFP expression. Prime editing can then be used to correct the mutated splice site and restore fluorescence in the cell. A Cas9 PAM sequence was identified 9 nt downstream of the *CTT* deletion on the template strand, with the 20 nt following on the coding strand making up the corresponding protospacer (Figure 3A). This PAM showed high *in silico* predicted cutting efficiency and proximity to the target mutation, while leaving room for an intact splice donor site flanking sequence (Figure 3A). This, combined with no immediate off-target sites, made it a promising candidate for use with the PEAR system. The pDas12124_PEAR-GFP-preedited plasmid with intact splice donor site was used as a starting point. A 3 bp deletion, found in F508del, was introduced into the splice site, removing the GT bases of the donor site as well as an additional base from the 5′ flanking sequence. This was done to ensure a low level of noise in GFP expression before editing. Downstream of the 4 bp 3′ flanking sequence, a 27 bp sequence was replaced with the sequence from genomic *CFTR*, including PAM sequence, protospacer as well as 4 bp for closer resemblance to the genomic target site. The upstream region of the newly introduced mutation contains the GFP coding sequence, which did not allow for major modification. Still, to emulate the AT-rich region upstream of the genomic *CTT* deletion, four silent mutations were added (Figure 3A).

The flow cytometry analyses of the different prime editor systems pCMV-PE2 and pCMV-PE6c showed that the pCMV-PE6c system had a 1.55-time higher prime editing efficiency compared to pCMV-PE2 (Supplementary Figure S1).

To assess the relationship between pegRNA structure and prime editing efficiency, different pegRNA variants were examined by editing the constructed pPEAR_CFTR plasmid (Figure 3C) in CFBE-X cells. In the following, pegRNAs targeting the pPEAR_CFTR plasmid are referred to as pegRNA_PEAR. The different numerical suffixes indicate variations in their structure and are listed in Figure 3B. This experiment was also carried out in HEK293 cells (Supplementary Figure S2). The flow cytometry analysis demonstrated that pegRNA_PEAR_9 (PBS: 16 nt, RTT: 33 nt, no silent edits) exhibited the most significant prime editing efficiency, reaching approximately 6.5% fluorescent cells. Notably, its editing efficiency accounts for more than half of that observed in the positive control, highlighting its superior performance. Most other pegRNA_PEARs, except for pegRNA_PEAR_4 (PBS: 17 nt, RTT: 27 nt, silent edits) and pegRNA_PEAR_5 (PBS: 16 nt, RTT: 30 nt, no silent edits) with around 4.7% fluorescent cells, displayed relatively similar editing efficiencies (2.5%–3.6% fluorescent cells). Among all tested constructs, pegRNA_PEAR_11 (PBS: 17 nt, RTT: 33 nt, no silent edits) exhibited the least significant editing efficiency at approximately 1.6% fluorescent cells (Figure 3C). Overall, pegRNA_PEAR_9 achieves the most significant prime editing efficiency, thus identifying its architecture as the most promising for the *CFTR* F508del mutation. Therefore, pegRNA_PEAR_9 was subsequently used for further experiments.

### 3.2 LNP formulation enables transfection better than PEI and cargo-loaded LNPs show lower zeta potential and more uniform size distribution

To assess the LNP formulation, its physicochemical properties, including transfection efficiency, zeta potential, size distribution, and uniformity, were measured to evaluate the impact of different components and loading conditions.

**FIGURE 4:**
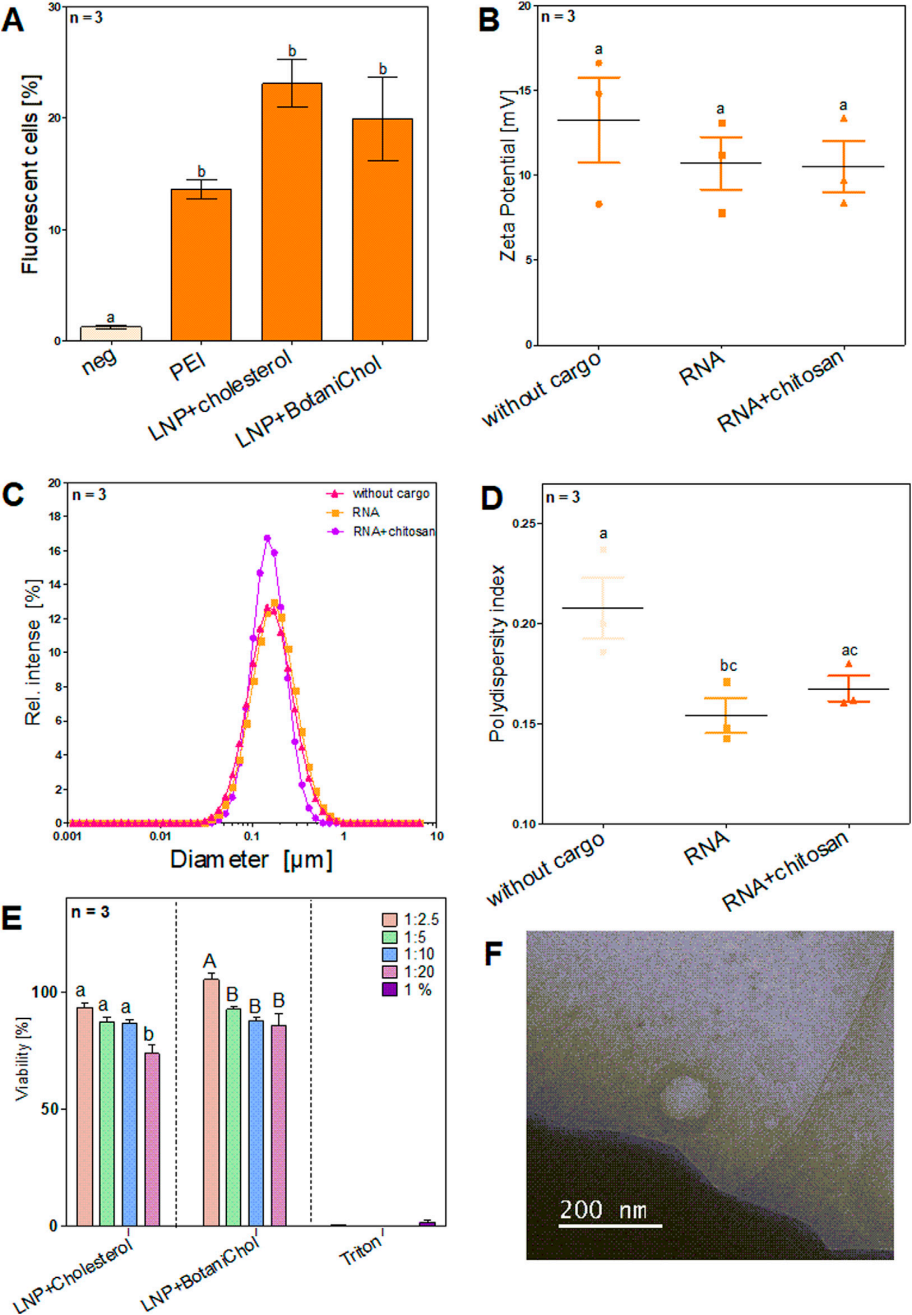
Characterization of LNP formulations and different cargo. Cells were seeded on 24-well plates at a density of 5 × 10^4^ cells/well prior to transfection and were then subjected to transfection at approximately 70% confluence. Cargo included 1,000 ng RNA per formulation. After cultivation cells were analyzed via flow cytometry and LNPs via Zetasizer **(A)** Comparison of Standard LNP formulation with cholesterol with plant based alternative BotaniChol, n = 3 biological replicates **(B)** Surface charge of LNPs with different or no cargo n = 3 technical replicates. **(C)** DLS analysis of LNP size n = 3 technical replicates. **(D)** Size distribution of LNPs with different or no cargo n = 3 technical replicates. **(E)** The cell viability (%) over 4 different time points (0 h, 2 h, 4 h, 6 h) for different LNP formulations at different dilutions (1:2.5, 1:5, 1:10, 1:20) and 1% Triton-X. Statistics were performed via two-way ANOVA with Tukey´s post hoc test. Within each graph, samples that differ significantly are indicated with a different letter and color (p < 0.05). Three biological replicates were measured. **(F)** Cryogenic transmission electron microscopy image of LNPs.

The percentage of fluorescent cells was measured to assess transfection efficiency across different formulations via flow cytometry (Figure 4A). PEI-based transfection resulted in a moderate increase in fluorescent cells, reaching approximately 10%. Among the LNP formulations, LNPs containing cholesterol exhibited the highest transfection efficiency, with over 20% fluorescent cells. LNPs formulated with BotaniChol showed a slightly lower transfection rate but remained comparable to the cholesterol-based LNPs. Error bars indicate variability within the replicates, with LNP-based formulations showing greater variation compared to PEI. All three transfection methods showed a significant difference compared to the negative control. LNPs without cargo exhibited the highest zeta potential, with a median of 14.5 mV (range: 8–16 mV). RNA loading resulted in a decrease, with a median zeta potential of ∼11 mV (range: 9–12 mV). The addition of chitosan to RNA-loaded LNPs led to a further slight reduction, yielding a median of ∼10 mV (range: 8–12 mV). Statistical analysis showed no significant difference between the three groups (Figure 4B).

The size distribution of LNP formulations was analyzed using DLS (Figure 4C). All formulations exhibited a similar size distribution, with a peak around 0.1 µm (∼100 nm). The relative intensity of scattered light was slightly higher for the RNA + chitosan formulation. No substantial shift in particle size was observed between formulations.

Analysis of the PDI revealed the highest value for LNPs without cargo, with a mean of ∼0.21, indicating greater heterogeneity. RNA-loaded LNPs exhibited a lower PDI (∼0.16), suggesting improved uniformity, while RNA + chitosan LNPs displayed a similar PDI (∼0.17) to the RNA-loaded group. Statistic analysis identified a significant difference in PDI between the LNP and LNP+RNA, whereas differences between LNP+RNA+Chitosan and LNP or LNP+RNA, despite seemingly present, were not significant. (Figure 4D).

The viability analysis revealed a largely constant cell survival rate in all tested conditions across all time points. Cell viability remained stable in all experimental groups and showed no significant deviations between the different time points (Supplementary Figures S3,S4). The non-loaded, cholesterol-containing LNPs showed significantly less viability when a 1:20 dilution was used. The non-loaded BotaniChol-containing LNPs showed significantly higher viability when 1:2.5 dilution was used. In contrast, the 1% Triton-X control led to a strongly reduced viability, as expected (Figure 4E). Moreover, the size of the LNP was determined using cryogenic transmission electron microscopy (Figure 4F). In summary, the results demonstrated that LNP formulations, particularly those containing cholesterol, achieved the highest transfection efficiency, significantly surpassing the negative control and PEI transfection. While Zeta potential and PDI measurements indicated slight variations, no significant differences were found between RNA and RNA + chitosan LNPs. Cell viability remained stable across all conditions, with notable exceptions for specific LNP dilutions, and cytotoxicity was observed in the 1% Triton-X control, as expected (Figure 4).

### 3.3 LNP-mediated delivery enhances transfection and prime editing efficiency in CFBE-X cells *in vitro*

**FIGURE 5:**
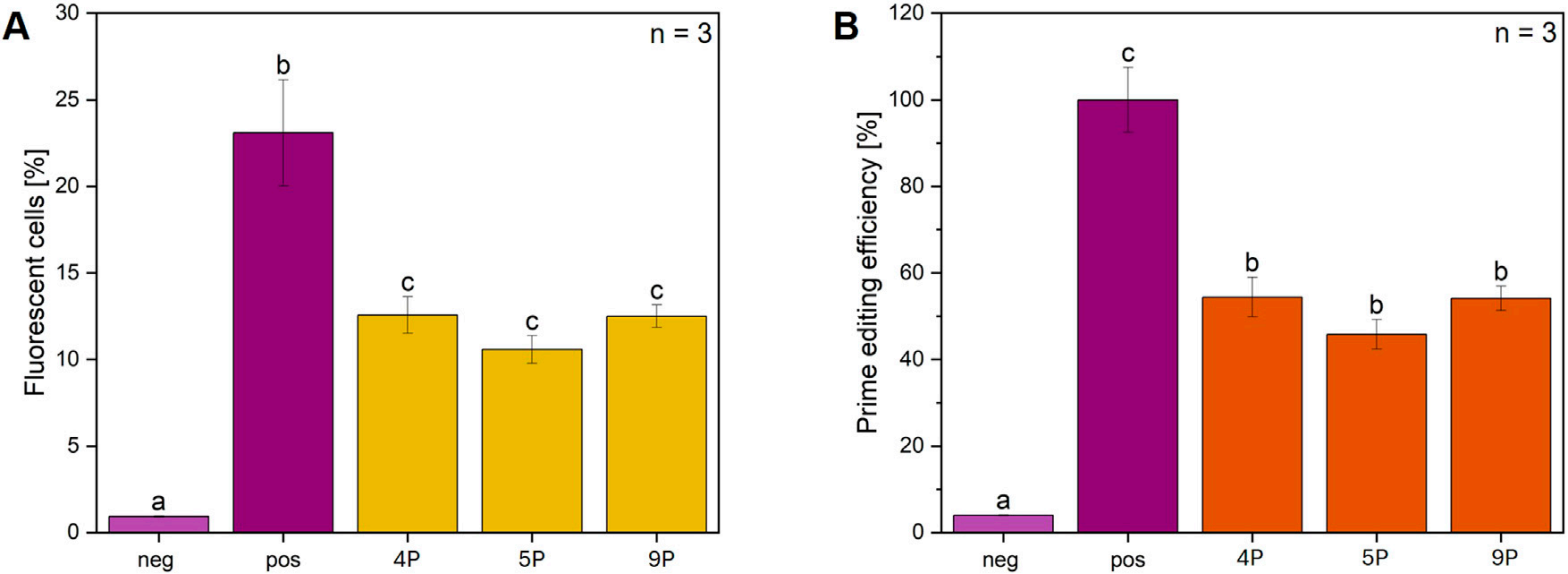
Delivery of prime editing components via LNPs: Flow cytometry analysis of CFBE-X cells with pPEAR-CFTR. **(A)** The percentage of fluorescent cells in the flow cytometry analysis is shown for three different pegRNAs (pegRNA 4, 5, and 9), which were selected and co-delivered with the pCMV-PE6c plasmid into CFBE-X cells using LNPs. The CFBE-X cells were initially transfected with pPEAR_CFTR, followed by the addition of LNPs loaded with pegRNA-chitosan-complex, n = 3 biological replicates. **(B)** The percentage of prime editing efficiency is shown for each pegRNA. All samples are shown for each condition compared to control, n = 3 biological replicates. Statistics were performed via one-way ANOVA with Tukey’s post hoc test. Within each graph, samples that differ significantly are indicated with a different letter and color (p < 0.05), “neg” denotes CFBE-X cells without transfection and “pos” cells transfected with a GFP expressing plasmid.

To enhance prime editing efficiency, chitosan and cholesterol complexed LNP were selected to target pPEAR-CFTR transfected in CFBE-X cells (Figure 5). The results show a significant increase in fluorescent cells for all three pegRNA_PEAR variants compared to control (Figure 5A). All tested pegRNA_PEAR constructs (pegRNA_PEAR_4, 5, and 9) resulted in values of fluorescent cells ranging between 10% and 13%. The highest fluorescence was observed for pegRNA_PEAR_4 and pegRNA 9, both reaching around 12%–13%, whereas pegRNA_PEAR_5 showed a lower efficiency of approximately 10%. Furthermore, the efficiency of prime editing was assessed and normalized to the positive control (Figure 5B). During this analysis, pegRNA_PEAR_4 and pegRNA_PEAR_9 exhibited comparable editing efficiencies of 56%, whereas pegRNA_PEAR_5 demonstrated a reduced efficiency of around 50%. Statistical analysis reveals no significant difference among the pegRNA_PEAR groups, suggesting comparable efficiency. As expected, the transfection efficiency increased by using LNPs by a factor of 1.5 compared to that of PEI tranfection.

### 3.4 Prime editing with pegRNA variants designed to correct the F508del genomic CFTR, verified by nanopore sequencing

**FIGURE 6:**
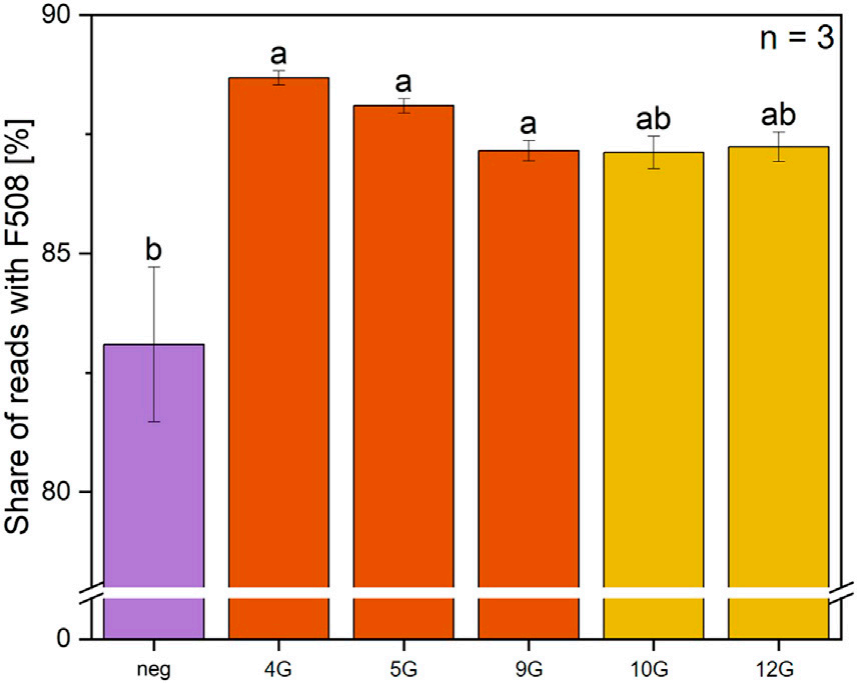
Sequencing analysis of prime editing gene efficiency for F508del correction using five distinct pegRNA variants. The percentage of sequencing reads with correct F508 is shown for prime editing with 5 different pegRNAs. PegRNA_genomic_CFTR_4, 5, 9, 10, and 12 were selected to target F508del and co-delivered with the pCMV-PE6c into the cells, n = 3. Statistics were performed via one-way ANOVA with Tukey’s post hoc test. Within each graph, samples that differ significantly are indicated with a different letter and color (p < 0.05).

In the next step, pegRNAs were specifically designed to target the *CFTR-*F508del region in the CFBE-X cell line, followed by a subsequent prime editing experiment and sequencing (Figure 6). Note that sequencing prior editing unexpectedly revealed, that the CFBE-X cell line carries the correct sequence in ∼83% of cases, and only ∼17% are editable. Again, the PE6c prime editor was used combined with the designed pegRNA_genomic_CFTR variants. The length of PBS and RTT and the silent edits were used as in the pegRNA_PEAR variants 4, 5, 9, 10 and 12 which showed the highest prime editing efficiency (Figure 3D). The genomic DNA of the cells was extracted and next-generation sequencing was used to analyze the percentage of sequencing reads containing the corrected F508 sequence. PegRNA_genomic_CFTR_4, 5, and 9 achieved the highest rates of correct DNA sequences, (∼85–88%), while pegRNA_genomic_CFTR_10 and 12 exhibit lower but comparable values (∼82–84%) (Figure 6).

## 4 Discussion

CF is a severe genetic disorder caused by mutations in the *CFTR* gene, leading to an imbalanced liquid homeostasis in lung epithelia cells. By aiming to optimize prime editing for the correction of the *CFTR* F508del mutation, a proof-of-concept pPEAR_CFTR-Reporter and pegRNAs were designed and co-delivered with pCMV-PE6c prime editor into CFBE-X cells using LNPs. Therefore, optimal LNP formulation was selected and LNPs were characterized. Transfection efficiency was evaluated using fluorescence reporters. Furthermore, genetic *CFTR* F508del was targeted, and prime editing efficiency was quantified via sequencing analysis.

### 4.1 Screening for optimal pegRNA variants in newly conceptualized reporter plasmid pPEAR-CFTR

The testing of pegRNA variants is of critical importance, as the pegRNA plays a decisive role, among other factors, in editing efficiency. It is important to note that the effectiveness of pegRNAs is highly context-dependent, meaning that there is not a universally optimal pegRNA. Instead, the pegRNA must be tailored to the specific application. To this end, a systematic testing of pegRNA variants was conducted using the constructed reporter system, with the objective of identifying the most suitable candidates for application within the given genomic context.

For precise genome editing, the selection of the spacer sequence in the pegRNA is critical, as it serves as the primary determinant of target specificity and potential off-target effects. To minimize such off-target activity, we used the CRISPick software (Doench et al., 2016) to evaluate candidate spacers and selected the one predicted to have the lowest off-target propensity. Furthermore, to increase stability, a structural motif was incorporated into the pegRNAs, which protects the pegRNAs from rapid degradation by ribonucleases, thereby increasing their lifespan in the cellular environment. Specifically, the tevopreQ1 stem loop was chosen, as established by Nelson et al. (2022). Since PBS and RTT lengths are highly context-dependent, several variants with different PBS and RTT lengths, as well as their combinations, were tested to determine the optimal length configuration for the target in question. We took into consideration that regions with a low GC content, as in this case (23.5% for a PBS length of 17 nt and 25% for a PBS length of 16 nt), typically require the use of longer PBS sequences, e.g., over 13 nt, for effective priming as shown by Anzalone et al., 2019. This is consistent with the energetic demands of the hybridization of the nicked DNA strand to the pegRNA PBS (Anzalone et al., 2019). Based on the results generated by the pegFinder software (Chow et al., 2021), variants with PBS lengths of 16 and 17 nt were tested. Additionally, six RT templates were evaluated, varying in length (27, 30 and 33 nt) and differing in the implementation of silent edits (being imperative to consider the silent edit leading to the PAM disrupt) – leading to a total of 12 pegRNA variants tested. It is noteworthy that RT templates which position a C adjacent to the 3-hairpin of the single guide RNA (sgRNA) scaffold generally result in reduced editing efficiency, so this was deliberately avoided when designing the pegRNAs (Anzalone et al., 2019). PegRNA variants with and without silent edits (Supplementary Table S3) were designed to evaluate their impact on editing efficiency and to determine whether such modifications improve or degrade performance.

The results show a strong dependence of prime editing efficiency on even small variations of pegRNA features. For example, pegRNA_PEAR_11 (PBS: 17 nt, RTT: 33 nt, no silent edits) and pegRNA_PEAR_12 (PBS: 17 nt, RTT: 33 nt, silent edits) yielded substantial differences in editing efficiency, varying from approximately 1.6%–4.0% fluorescent cells. Furthermore, no PBS or RTT length was strictly predictive of editing efficiency, suggesting that other factors such as RT template secondary structure also influence editing activity (Figure 3B). These findings are consistent with those previously reported by Anzalone et al., 2019, thereby providing further support for the conclusion.

The incorporation of silent edits leads to enhanced editing efficiency when using an RTT length of 27 nucleotides. However, this effect is not consistently observed across all RT lengths. For other pegRNA variants, the presence of silent edits does not necessarily improve editing efficiency and, in some cases, even reduces it. This is particularly evident in the case of pegRNA_PEAR_9 (PBS: 16 nt, RTT: 33 nt, no silent edits), which exhibits the highest editing efficiency without silent edits while showing a 1.8-fold increase in editing efficiency compared to pegRNA_PEAR_10 (PBS: 16 nt, RTT: 33 nt, silent edits). The findings suggest that the impact of silent edits is also context-dependent and influenced by the specific RT template length (Figure 3C). The conclusions drawn by Li et al., 2022, who reported a substantial enhancement in efficiency for base editing, up-to 4,976-fold, through the implementation of same-sense mutations led to the formulation of a hypothesis that predicts a comparable effect for multi-base edits. However, our data do not support this hypothesis. Consequently, further research is necessary, particularly concerning the evaluation of various silent edits and their combinations.

### 4.2 Characterization of lung specific LNP formulation with RNA-chitosan-complexation and plant-based cholesterol alternative

In the context of gene delivery, it is well-established that the efficiency of transfection is influenced by the formulation and the resulting cellular uptake and endosomal escape of the delivery system. As PEI, a widely used non-viral transfection method, has been associated with high cytotoxicity and suboptimal biocompatibility depending on molecular size (Moghimi et al., 2005), we investigated delivery via LNPs. Our results show that the tested LNP formulations achieve a higher transfection rate compared to that of the PEI control but without significant cytotoxicity (Figures 4A,E). In LNP formulations PEGylation is an important factor providing stability, solubility and stealth functionality by preventing aggregation, unintended adherence, and detection by immune cells. However, it has been shown that excessive PEG modification hinders transfection efficiency by reducing cellular uptake and impeding the endosomal escape of the complexes (Binici et al., 2025). Our results did not show reduced transfection rates although the PEG concentration with molar 2.7 % in our formulation was high. This suggests that in our LNP formulation and used transfection conditions, the increased PEG content did not have a negative effect on the transfection rate (Figure 4A).

Another important ingredient in LNP formulation is cholesterol, since it is critical for stabilizing the lipid layer, particle formation or enhancing the efficiency of gene transfection by facilitating the endosomal escape (Zidovska et al., 2009). In addition, studies demonstrated that cholesterol analogs enhanced transfection rates compared to standard cholesterol, as they improve the polymorphic shape of lipid nanoparticles and enhance intracellular mRNA delivery (Patel et al., 2020). We explored the use of a plant-derived cholesterol as a vegan alternative to conventional animal-based cholesterol. For that, we evaluated a plant-derived cholesterol named ‘BotaniChol’, which is labeled as >99% pure. As expected, the results showed no significant change in transfection efficiency (Figure 4A) and there were no notable differences in cytotoxicity between the two formulations (Figure 4E). This suggests that plant-based cholesterol may be used as a replacement.

Zeta potential analysis showed no significant differences among the three tested groups, suggesting that neither the presence of cargo nor chitosan complexation substantially alters surface charge (Figure 4B). Despite the incorporation of the cationic lipid 4A3 SC8 and 39.4% DOTAP, which would typically result in a more positively charged LNP, our formulation exhibited a relatively low zeta potential between 10 and 13 mV. This can possibly be attributed to the presence of the high PEG concentration in our LNP formulation, which likely shields surface charge and influences zeta potential measurements, a phenomenon previously reported in the literature (Choi et al., 2025).

Distinct differences were observed in PDI. LNPs without cargo exhibited a higher PDI and a broader distribution range, suggesting greater variability in size distribution. Conversely, LNPs loaded with RNA, as well as those containing RNA-chitosan complexes, showed a lower mean PDI and a more uniform size distribution (Figure 4D). This indicates that the incorporation of cargo facilitates the formation of LNPs with consistent size. While the addition of chitosan influenced the size distribution, the observed differences between RNA-loaded LNPs with and without chitosan were not significant (Figure 4D).

These findings highlight the importance of optimizing LNP composition to balance stability, transfection efficiency, and homogeneity. Further investigations should focus on refining PEG content and exploring alternative strategies to enhance endosomal escape while maintaining effective cellular uptake stability. Despite these promising results, several challenges remain. An important factor for the potential application of LNP-mediated gene therapy in CF patients that still requires thorough evaluation is the impact of the viscous mucus accumulation that occurs *in vivo* on the therapeutic effectiveness. While this study focused on optimizing the LNP formulation for compatibility with the therapeutic mRNA and a lung cell line, the formulation needs to be further optimized for *in vivo* application. In addition to the affinity of the LNPs to the mucus, the size is also a key factor in mucus penetration. While larger LNPs may bind to the mucus but not necessarily pass through it, smaller LNPs (up to 500 nm) can efficiently pass through the barrier and reach the epithelial lining (Zhao et al., 2022). With a diameter of just over 100 nm (Figure 4C), our LNPs showed the optimal prerequisite for overcoming the mucus barrier.

Prior to the administration of LNP-based therapeutics via inhalation, the airway mucus barrier can be modulated to facilitate particle penetration. In CF patients, the mucus layer is pathologically thickened and exhibits increased viscoelasticity, impeding the diffusion of inhaled nanoparticles toward the epithelial surface. Inhalation of hypertonic saline has been demonstrated to exert mucolytic effects and to enhance mucociliary clearance by hydrating and partially restoring the periciliary liquid layer lining the airways (Reeves et al., 2012). Another way to make mucus more penetrable is to use the mucolytic properties of N-acetylcysteine. This active ingredient is already used for inhalation in CF patients and, in addition to its mucolytic properties, also has antioxidant and antibiofilm benefits (Guerini et al., 2022). Administering hypertonic saline or N-acetylcysteine before LNP inhalation is anticipated to transiently reduce mucus viscosity and improve its permeability, thereby enabling more efficient traversal of the mucus mesh by LNPs and enhancing their subsequent interaction with target epithelial cells.

In subsequent experimental stages, the LNP formulation could be further engineered to achieve selective delivery to basal epithelial cells within the airway epithelium through a dual-display surface strategy. In this design, one moiety would serve as a transcytosis ligand, such as albumin, to mediate passage through the overlying epithelial cell layer via receptor-dependent vesicular transport. The second moiety would be a basal cell–targeting ligand, for example through targeting basal cell markers like Trp-63 (p63) and cytokeratin 5 and 14 (Krt5/14), to promote preferential uptake by basal cells through receptor-mediated endocytosis (Rock et al., 2009). This combinatorial targeting approach would enable the LNPs to traverse the differentiated epithelial layer intact and subsequently bind to and be internalized by basal cells, where the therapeutic payload would be released following endosomal escape.

### 4.3 Using optimized pegRNAs for efficient prime editing of both pPEAR_CFTR and the genomic CFTR locus, delivered via LNPs

The efficiency of prime editing depends on various factors that can be specifically optimized. For example, the impact of pegRNA design on editing efficiency demonstrates that even small sequence variations can lead to significant differences. While certain trends, such as the influence of silent edits and PBS/RTT lengths, were observed, optimal pegRNA design remains highly context dependent. The PE6c system with pegRNA_PEAR_4 or pegRNA_PEAR_9, mediated by LNP and chitosan, achieved a prime editing efficiency of 54% in pPEAR_CFTR transfected CFBE-X cells (Figure 5B) while the use of LNPs resulted in approximately twice the transfection efficiency compared to transfection without LNPs (Figure 4A). This demonstrates that LNPs significantly enhance the uptake and expression of the delivered components. To put the results into perspective, similar efficiencies of over 53% were achieved in HEK293-Gal9-GFP cells using LNPs loaded with PE3 and pegRNA (Herrera-Barrera et al., 2023). Additionally, PE6c-mediated correction of the pathogenic RECQL3 (Bloom Syndrome) mutation at nt 2,281 (6 bp deletion/7 bp insertion) in HEK293T cells resulted in a prime editing efficiency of nearly 32% without detectable indels (Doman et al., 2023). Furthermore, the efficiency of prime editing is not only determined by the pegRNA design, but also influenced by the cellular environment, the transfection method and the prime editing system used. In the context of prime editing, the pPEAR_CFTR model provides proof of concept for the targeted correction of F508del mutation. Through targeted screening experiments, these variables can be systematically optimized to adapt prime editing to specific cell types and therapeutic applications.

The final experiment in this study targeted the F508del mutation in the *CFTR* gene. For this approach, the PE6c prime editor was used alongside the pegRNA variants that showed the highest prime editing efficiency with the reporter system. In these preliminary experiments, the best RTT and PBS lengths and the use of silent edits were investigated. The analysis shows that the use of pegRNA_genomic_CFTR_4, 5, and 9 resulted in a significant proportion of reads with the correct F508 sequence (Figure 6).

Contrary to the expectation that the F508del mutation is homozygous in the cell line, the negative control showed the corrected wild type sequence with CTT in approximately 83% of the reads. Accounting for these results, the total amount of editable genes is only 17%. The percentage of edited cells is around 5%, if the proportion of genes that were previously correct is subtracted. This is the same percentage range as found in the literature (Sousa et al., 2024), where between 2% and 15% of sequencing reads were found to contain the CTT insertion. To make rough statements about the primary editing efficiency, it is necessary to consider the transfection efficiency. With the conditions and cells used in this study the transfection efficiency is between 10% and 15%. By calculating the number of edited cells divided per transfection rate, it could be assumed that the prime editing efficiency is around 40%. Again, pegRNA_genomic_CFTR_4 and 5 showed higher prime editing efficiency than the other three tested variants.

Targeted gene correction is a promising approach for treating CF by directly addressing its genetic cause. The pPEAR_CFTR model in CFBE-X cell line demonstrates that prime editing can precisely correct mutations in the *CFTR* gene like model. With further modifications basal lung cells could be targeted with even higher efficiency. The results of the *in vitro* experiments must undergo extensive *in vivo* experiments in a suitable animal model to validate the results. This is needed to ensure efficacy and security of the system. This also includes the investigation of potential long-term effects as well as possible side effects of the therapy. The implementation into a clinical application will depend on these results (European Medicines Agency, 2018).

## Data Availability

The original contributions presented in the study are included in the article/Supplementary Material, further inquiries can be directed to the corresponding author.

## Supplementary material

SUPPLEMENTARY FIGURE S1 Flow cytometry measurements of edited HEK293 cells, 100,000 counts in one experiment. Comparison of mean values of different Prime Editors (PE2 and PE6c). The negative control (light purple) consists of untreated cells, the positive control (purple) includes cells transfected with pZMB0938, sample cells were transfected with pDAS12489 and either pCMV-PE2 (light blue) or pCMV-PE6c (dark blue).

SUPPLEMENTARY FIGURE S2 Flow cytometry measurements of edited HEK293 cells containing the pPEAR_CFTR plasmid, 100,000 counts in one experiment. Comparison of mean values of different pegRNAs varying in their architecture regarding the Primer binding sequence (PBS), Reverse transcriptase template (RTT) and incorporation (+) or not (‐) of silent edits. The negative control (light purple) consists of untreated cells, the positive control (purple) includes cells transfected with pZMB0938 and sample cells were transfected with pPEAR_CFTR and pU6-pegRNA-GG-acceptor plasmid containing the different pegRNAs (yellow).

SUPPLEMENTARY FIGURE S3 The viability of cholesterol-containing non-loaded LNP with 4 different dilutions (1:2.5, 1:5, 1:10, 1:20) and 1% Triton was measured at 4 different time points (0 h, 2 h, 4 h and 6 h). Statistics were performed via two-way ANOVA with Tukey´s post hoc test. 3 biological replicates were measured.

SUPPLEMENTARY FIGURE S4 The viability of bota-containing non-loaded LNP with 4 different dilutions (1:2.5, 1:5, 1:10, 1:20) and 1% Triton was measured at 4 different time points (0 h, 2 h, 4 h and 6 h). Statistics were performed via two-way ANOVA with Tukey´s post hoc test. 3 biological replicates were measured.
