# Supplementary material for: Towards effective cystic fibrosis gene therapy by optimizing prime editing and pulmonary-targeted LNPs
Source: Front Syst Biol. 2025 Dec 3;5:1603749. doi: 10.3389/fsysb.2025.1603749 (PMC12710416; doi:10.3389/fsysb.2025.1603749)
Supplement: Supplementary file 1 [file Supplementaryfile1.docx]

Supplementary Material

Towards effective cystic fibrosis gene therapy by optimizing prime editing and pulmonary-targeted LNPs

# Supplementary Figures

##
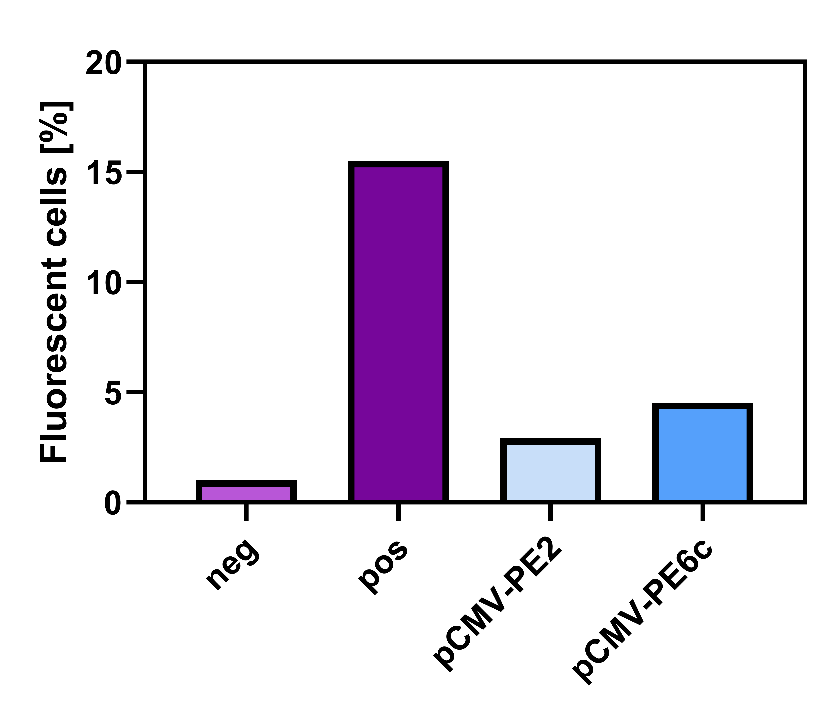


**Supplementary Figure 1.** Flow cytometry measurements of edited HEK293 cells, 100,000 counts in one experiment. Comparison of mean values of different Prime Editors (PE2 and PE6c). The negative control (light purple) consists of untreated cells, the positive control (purple) includes cells transfected with pZMB0938, sample cells were transfected with pDAS12489 and either pCMV-PE2 (light blue) or pCMV-PE6c (dark blue).


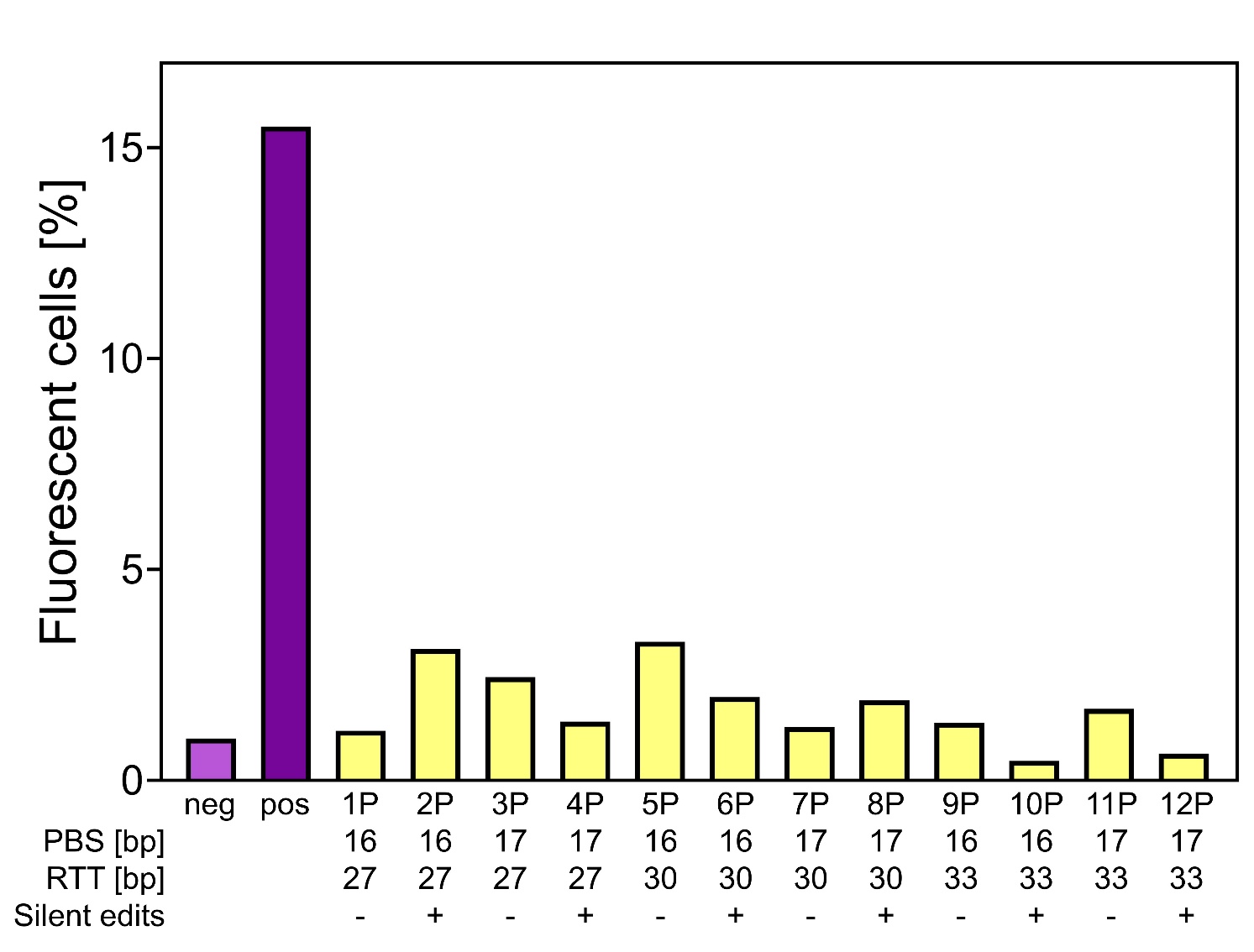


**Supplementary Figure 2.** Flow cytometry measurements of edited HEK293 cells containing the pPEAR_CFTR plasmid, 100,000 counts in one experiment. Comparison of mean values of different pegRNAs varying in their architecture regarding the Primer binding sequence (PBS), Reverse transcriptase template (RTT) and incorporation (+) or not (-) of silent edits. The negative control (light purple) consists of untreated cells, the positive control (purple) includes cells transfected with pZMB0938 and sample cells were transfected with pPEAR_CFTR and pU6-pegRNA-GG-acceptor plasmid containing the different pegRNAs (yellow).


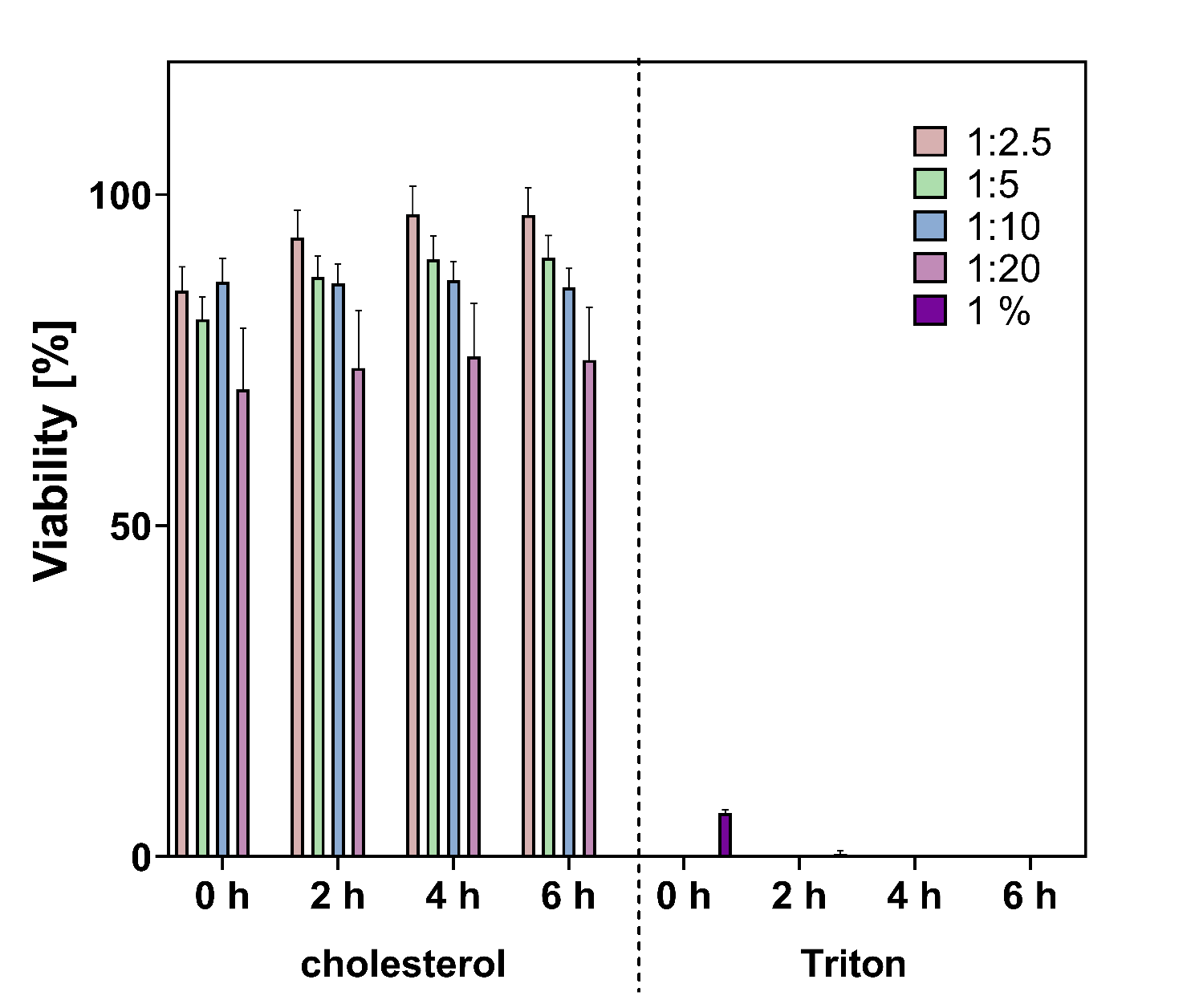


**Supplementary Figure 3.** The viability of cholesterol-containing non-loaded LNP with four different dilutions (1:2.5, 1:5, 1:10, 1:20) and 1 % Triton was measured at four different time points (0 h, 2 h, 4 h and 6 h). Statistics were performed via two-way ANOVA with Tukey´s post hoc test. Three biological replicates were measured.


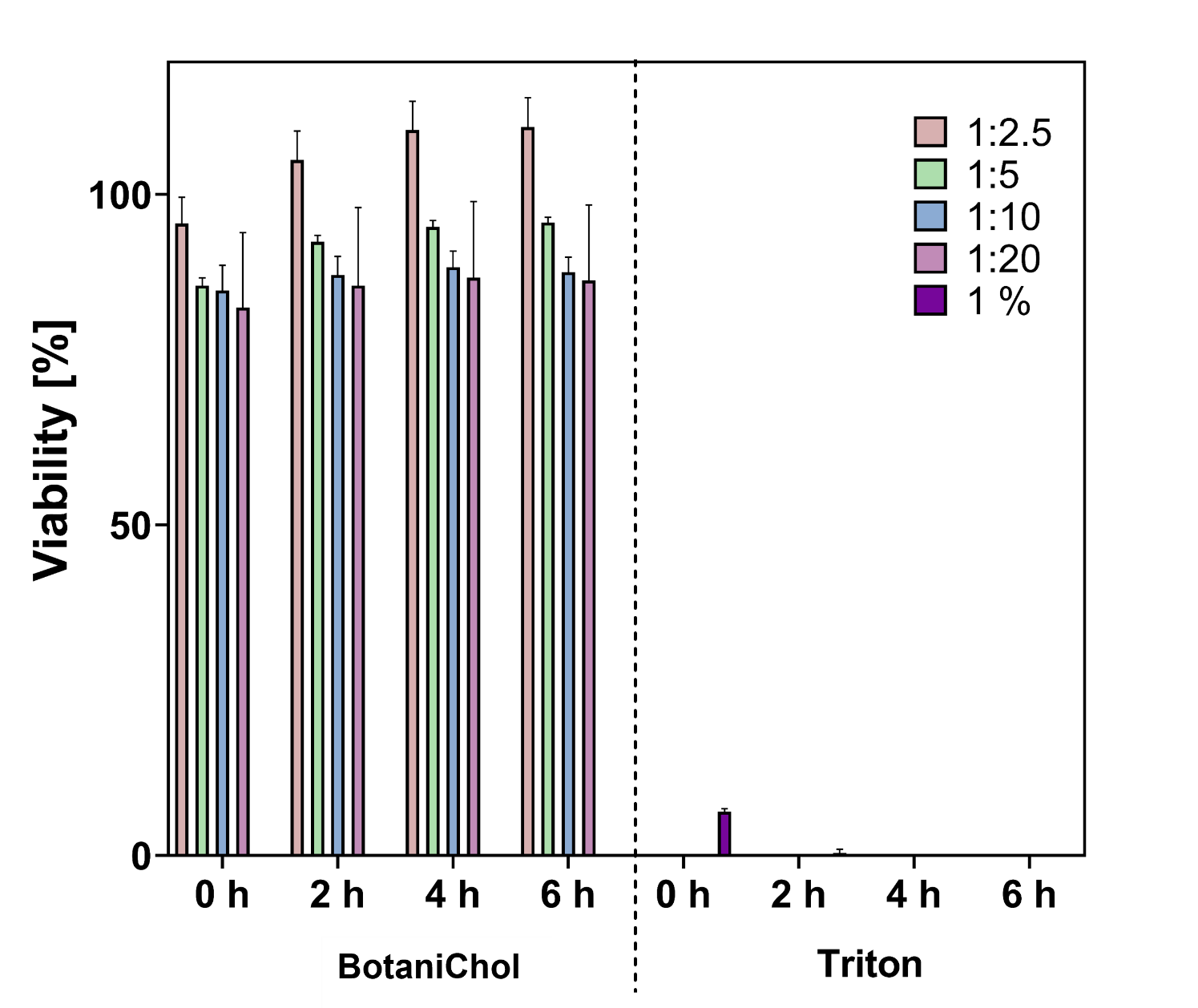


**Supplementary Figure 4.** The viability of BotaniChol-containing non-loaded LNP with four different dilutions (1:2.5, 1:5, 1:10, 1:20) and 1 % Triton was measured at four different time points (0 h, 2 h, 4 h and 6 h). Statistics were performed via two-way ANOVA with Tukey´s post hoc test. Three biological replicates were measured.

# Supplementary Tables

**All supplementary tables are included in the file Suplementary_data.xlsx submitted as supplementary data.**

**Supplementary Table 1.** Overview of ordered plasmids, gene syntheses, and derived cloned constructs. The table provides a summary of all ordered plasmids and gene synthesis products, as well as the resulting cloned constructs generated from these materials. Further details include antibiotic resistance, construct size, relevant restriction sites, and a brief description of each entry.

**Supplementary Table 2.** Overview of all relevant primers that have been utilized for the context of cloning and sequencing. It includes additional information on their specific applications and details including GC content, melting temperature, annealing temperature and the corresponding products generated.

**Supplementary Table 3.** Summarization of all designed pegRNA oligonucleotides, providing comprehensive details on their architectural composition, length, and sequence.

**Supplementary Table 4.** Raw data of the flow cytometry analysis of CFBE-X transfected with pPEAR‑CFTR, pCMV-PE6c and pegRNA variants, this data is displayed in figure 3 in the manuscript.

**Supplementary Table 5.** Raw data LNP characterization of the different methods, this data is displayed in figure 4 in the manuscript.

**Supplementary Table 6.** Raw data of the flow cytometry analysis of CFBE-X transfected with pPEAR‑‑CFTR and pCMV-PE6c and pegRNA variants delivered with LNPS, this data is displayed in figure 5 in the manuscript.

**Supplementary Table 7.** Raw data of the sequencing analysis of CFBE-X transfected with pCMV-PE6c and pegRNA variants, this data is displayed in figure 6 in the manuscript.
